# Supplementary material for: Transcriptome and Metabolome Reveal the Molecular Mechanism of Barley Genotypes Underlying the Response to Low Nitrogen and Resupply
Source: Int J Mol Sci. 2023 Feb 28;24(5):4706. doi: 10.3390/ijms24054706 (PMC10003240; doi:10.3390/ijms24054706)
Supplement: Supplementary file 1 [file ijms-24-04706-s001.zip › ijms-2118769-supplementary.pdf]

Table S1. Summary of data quality for Agilent 2100 bioanalyzer RNA-Seq

Sample name	Raw reads	Clean reads	clean bases	Error rate(%)	Q20(%)	Q30(%)	GC content(%)
d3W20CK-R1	47788704	45953730	6.89G	0.03	97.84	94	52.75
d3W20CK-R2	46933016	46851478	7.03G	0.03	96.88	91.81	47.3
d3W20CK-R3	47800050	45674520	6.85G	0.03	97.62	93.85	53.6
d3W20CK-S1	45523156	44155838	6.62G	0.03	97.33	92.92	56.03
d3W20CK-S2	47629842	46002256	6.9G	0.03	97.28	92.84	55.7
d3W20CK-S3	44995338	42753996	6.41G	0.03	97.45	93.16	55.56
d3W20LN-R1	45786504	42605640	6.39G	0.03	97.17	92.54	49.09
d3W20LN-R2	45528478	41118650	6.17G	0.03	97.71	94.15	52.45
d3W20LN-R3	47348076	44111894	6.62G	0.03	97.63	94.05	53.74
d3W20LN-S1	45538286	42721164	6.41G	0.03	98.07	94.02	53.86
d3W20LN-S2	44307084	40028848	6.0G	0.03	96.85	92.1	53.71
d3W20LN-S3	44153486	40643100	6.1G	0.03	96.57	91.63	54.21
d18W20CK-R1	46545152	43414066	6.51G	0.03	97.78	94.28	55.58
d18W20CK-R2	46445930	45862768	6.88G	0.03	96.75	91.6	54.17
d18W20CK-R3	49540452	48885680	7.33G	0.03	96.52	91.05	54.2
d18W20CK-S1	46218910	46108650	6.92G	0.03	97.8	94.37	58.02
d18W20CK-S2	47623686	43592746	6.54G	0.03	97.63	94.01	57.49
d18W20CK-S3	46200204	41699948	6.25G	0.03	96.87	92.05	55.31
d18W20LN-R1	44301262	41613304	6.24G	0.03	96.66	91.49	54.01
d18W20LN-R2	42636508	42077718	6.31G	0.03	96.74	91.55	53.35
d18W20LN-R3	40835218	40280280	6.04G	0.03	96.76	91.64	53.31
d18W20LN-S1	47735060	43890512	6.58G	0.03	96.75	91.76	54.74
d18W20LN-S2	46125002	43199138	6.48G	0.03	96.64	91.48	55.92
d18W20LN-S3	44580348	42228796	6.33G	0.03	96.72	91.76	55.45
d21W20LN-R1	45247544	43112342	6.47G	0.03	96.75	91.61	54.63
d21W20LN-R2	44478884	42213122	6.33G	0.03	96.86	91.87	56.04
d21W20LN-R3	43860530	41601296	6.24G	0.03	96.87	92.04	54.41
d21W20LN-S1	44128358	40673630	6.1G	0.03	97	92.12	54.9
d21W20LN-S2	50274638	47553822	7.13G	0.03	96.76	91.69	57.12
d21W20LN-S3	47003056	44179902	6.63G	0.03	96.71	91.64	56.6
d21W20RN-R1	43758642	41710292	6.26G	0.03	96.61	91.5	54.51
d21W20RN-R2	47055718	46446830	6.97G	0.03	96.83	91.74	54.18
d21W20RN-R3	46679828	46067052	6.91G	0.03	96.23	90.52	54.31
d21W20RN-S1	47667426	44517500	6.68G	0.03	96.95	92.18	58.04
d21W20RN-S2	45410074	43036218	6.46G	0.02	97.82	94.42	57.98
d21W20RN-S3	43447504	41302634	6.2G	0.03	96.76	91.79	57.68
d3W26CK-R1	44932028	42819102	6.42G	0.03	96.83	91.75	54.84
d3W26CK-R2	46114032	45490322	6.82G	0.03	96.54	91.18	54.11
d3W26CK-R3	46576936	44652684	6.7G	0.02	98.02	94.31	51.84
d3W26CK-S1	46042808	43421708	6.51G	0.03	96.51	91.38	56.33
d3W26CK-S2	44901530	41452298	6.22G	0.03	96.75	91.77	55.88
d3W26CK-S3	45274838	41924150	6.29G	0.03	96.78	91.79	56.72
d3W26LN-R1	45534808	41570218	6.24G	0.03	96.81	91.75	54.84
d3W26LN-R2	54405074	50273794	7.54G	0.03	97.62	93.86	54
d3W26LN-R3	46477686	45844074	6.88G	0.03	96.51	91.07	53.34
d3W26LN-S1	45972962	42918006	6.44G	0.03	96.36	90.75	55.12
d3W26LN-S2	43772414	40621834	6.09G	0.03	97.63	94.05	54.18

d3W26LN-S3	45670474	42865350	6.43G	0.03	97.06	92.53	54.32
d18W26CK-R1	47175252	44377344	6.66G	0.03	96.82	91.65	54.36
d18W26CK-R2	45852636	43871572	6.58G	0.03	97.88	94.24	54.24
d18W26CK-R3	42370622	40421444	6.06G	0.03	96.64	91.33	53.51
d18W26CK-S1	55398722	52892072	7.93G	0.03	96.62	91.41	57.63
d18W26CK-S2	44246176	41921060	6.29G	0.03	97.77	94.28	57.31
d18W26CK-S3	44497444	42457454	6.37G	0.03	96.46	91.24	58.66
d18W26LN-R1	46948304	44255874	6.64G	0.03	97.35	93.3	52.16
d18W26LN-R2	48484330	44218922	6.63G	0.03	96.75	91.63	56.32
d18W26LN-R3	47635334	46440160	6.97G	0.03	96.75	91.65	54.6
d18W26LN-S1	46862456	43186012	6.48G	0.03	96.64	91.5	55.84
d18W26LN-S2	45324082	41808746	6.27G	0.03	96.57	91.46	56.48
d18W26LN-S3	44656508	41818748	6.27G	0.03	96.69	91.53	55.53
d21W26LN-R1	45963458	42738802	6.41G	0.03	97.56	93.91	55.76
d21W26LN-R2	45502218	42296272	6.34G	0.03	96.85	91.95	55.71
d21W26LN-R3	43104456	40440998	6.07G	0.03	96.69	91.41	55.75
d21W26LN-S1	45618900	43213086	6.48G	0.03	97.62	94.03	56.55
d21W26LN-S2	42303066	40117052	6.02G	0.03	96.65	91.44	57.62
d21W26LN-S3	45669722	43650880	6.55G	0.03	96.7	91.53	58.59
d21W26RN-R1	45592246	43173638	6.48G	0.03	96.8	91.61	54.52
d21W26RN-R2	47735188	45760250	6.86G	0.03	97.92	94.24	53.99
d21W26RN-R3	48044882	44961994	6.74G	0.02	97.84	94.44	54.41
d21W26RN-S1	48123080	44478694	6.67G	0.03	96.68	91.62	58.55
d21W26RN-S2	45786792	42334038	6.35G	0.03	97.04	92.2	58.76
d21W26RN-S3	46548518	43253662	6.49G	0.03	96.79	91.85	57.86

Table S2. Summary of the sequencing data generated for RNA-Seq and mapping on the barley genome.

Sample	Total reads	Total map	Unique map (%)
d3W20CK-R1	45953730	42132080(91.68%)	41389901(90.07%)
d3W20CK-R2	46851478	43010683(91.8%)	42559440(90.84%)
d3W20CK-R3	45674520	41125557(90.04%)	40369478(88.39%)
d3W20CK-S1	44155838	41526225(94.04%)	39343791(89.1%)
d3W20CK-S2	46002256	43172407(93.85%)	40936824(88.99%)
d3W20CK-S3	42753996	40175849(93.97%)	38040162(88.97%)
d3W20LN-R1	42605640	35238510(82.71%)	34826739(81.74%)
d3W20LN-R2	41118650	35431763(86.17%)	34815926(84.67%)
d3W20LN-R3	44111894	34105573(77.32%)	33539763(76.03%)
d3W20LN-S1	42721164	39947167(93.51%)	38479764(90.07%)
d3W20LN-S2	40028848	35970390(89.86%)	34734178(86.77%)
d3W20LN-S3	40643100	36778844(90.49%)	35567118(87.51%)
d18W20CK-R1	43414066	34394633(79.22%)	33548438(77.28%)
d18W20CK-R2	45862768	36499216(79.58%)	35532013(77.47%)
d18W20CK-R3	48885680	38883219(79.54%)	37860835(77.45%)
d18W20CK-S1	46108650	42938845(93.13%)	41482593(89.97%)
d18W20CK-S2	43592746	40599476(93.13%)	39496072(90.6%)
d18W20CK-S3	41699948	38689231(92.78%)	37989082(91.1%)
d18W20LN-R1	41613304	29496822(70.88%)	29018715(69.73%)
d18W20LN-R2	42077718	30453342(72.37%)	29930541(71.13%)
d18W20LN-R3	40280280	29108489(72.26%)	28607701(71.02%)
d18W20LN-S1	43890512	40607571(92.52%)	39425857(89.83%)
d18W20LN-S2	43199138	40046932(92.7%)	39340598(91.07%)
d18W20LN-S3	42228796	38959705(92.26%)	38263630(90.61%)
d21W20LN-R1	43112342	31838274(73.85%)	31124108(72.19%)
d21W20LN-R2	42213122	36823225(87.23%)	36090126(85.5%)
d21W20LN-R3	41601296	33944220(81.59%)	33316303(80.08%)
d21W20LN-S1	40673630	37609268(92.47%)	36016915(88.55%)
d21W20LN-S2	47553822	43488481(91.45%)	41074449(86.37%)
d21W20LN-S3	44179902	40919498(92.62%)	38571503(87.31%)
d21W20RN-R1	41710292	29894644(71.67%)	29258293(70.15%)
d21W20RN-R2	46446830	34293530(73.83%)	33500081(72.13%)
d21W20RN-R3	46067052	33822783(73.42%)	33061919(71.77%)
d21W20RN-S1	44517500	41573070(93.39%)	39007638(87.62%)
d21W20RN-S2	43036218	40340796(93.74%)	38616413(89.73%)
d21W20RN-S3	41302634	38252598(92.62%)	36751864(88.98%)
d3W26CK-R1	42819102	35593779(83.13%)	34951172(81.63%)
d3W26CK-R2	45490322	38575258(84.8%)	37853147(83.21%)
d3W26CK-R3	44652684	32507634(72.8%)	31852008(71.33%)
d3W26CK-S1	43421708	39998133(92.12%)	38100609(87.75%)
d3W26CK-S2	41452298	38440814(92.74%)	36804770(88.79%)
d3W26CK-S3	41924150	38639760(92.17%)	36632279(87.38%)
d3W26LN-R1	41570218	31415768(75.57%)	30851890(74.22%)
d3W26LN-R2	50273794	44099679(87.72%)	43323092(86.17%)
d3W26LN-R3	45844074	40204430(87.7%)	39465614(86.09%)
d3W26LN-S1	42918006	39581228(92.23%)	38176324(88.95%)

d3W26LN-S2	40621834	37748646(92.93%)	36485748(89.82%)
d3W26LN-S3	42865350	39750702(92.73%)	38488936(89.79%)
d18W26CK-R1	44377344	33804598(76.18%)	32982596(74.32%)
d18W26CK-R2	43871572	34698596(79.09%)	33853986(77.17%)
d18W26CK-R3	40421444	33111468(81.92%)	32578312(80.6%)
d18W26CK-S1	52892072	49075767(92.78%)	47338514(89.5%)
d18W26CK-S2	41921060	39135107(93.35%)	37762678(90.08%)
d18W26CK-S3	42457454	39105484(92.11%)	37534637(88.41%)
d18W26LN-R1	44255874	38864388(87.82%)	38149366(86.2%)
d18W26LN-R2	44218922	36368143(82.25%)	35672989(80.67%)
d18W26LN-R3	46440160	38863660(83.69%)	38075440(81.99%)
d18W26LN-S1	43186012	39814845(92.19%)	38726072(89.67%)
d18W26LN-S2	41808746	38321700(91.66%)	37323919(89.27%)
d18W26LN-S3	41818748	38392437(91.81%)	37581528(89.87%)
d21W26LN-R1	42738802	35186530(82.33%)	34287079(80.22%)
d21W26LN-R2	42296272	33550086(79.32%)	32661178(77.22%)
d21W26LN-R3	40440998	32185463(79.59%)	31431138(77.72%)
d21W26LN-S1	43213086	40012334(92.59%)	39011701(90.28%)
d21W26LN-S2	40117052	36972366(92.16%)	35634580(88.83%)
d21W26LN-S3	43650880	40534871(92.86%)	38727796(88.72%)
d21W26RN-R1	43173638	30823810(71.39%)	30156307(69.85%)
d21W26RN-R2	45760250	35601881(77.8%)	34839224(76.13%)
d21W26RN-R3	44961994	40527911(90.14%)	39848834(88.63%)
d21W26RN-S1	44478694	40937165(92.04%)	39047177(87.79%)
d21W26RN-S2	42334038	39292585(92.82%)	37513810(88.61%)
d21W26RN-S3	43253662	40036915(92.56%)	38237285(88.4%)

Table S3. Candidate genes related to different N treatment in barley nitrogen metabolism in leaves.

Gene ID	W26 Log$_2$FC			W26 Log$_2$FC			Annotation
	3d LN/CK	19d LN/CK	22d LN/R	3d LN/CK	19d LN/CK	22d LN/R	
**Nitrite transporter**							
HORVU.MOREX.r3.7HG0700030				-1.1551			Nitrate transporter 1.1
HORVU.MOREX.r3.7HG0696870					-3.8935		Nitrate transporter 1.3
HORVU.MOREX.r3.3HG0255820				-4.7821			Nitrate transporter At3g43790
HORVU.MOREX.r3.6HG0609530	4.1642	2.7768			-3.7077		Nitrate transporter 1.5
HORVU.MOREX.r3.4HG0334860				2.1568			Nitrate transporter 1.10
HORVU.MOREX.r3.6HG0605270				3.9590			Nitrate transporter 1.8
HORVU.MOREX.r3.5HG0510530	2.3096						Nitrate transporter 1.2
HORVU.MOREX.r3.2HG0155590	2.1490						Nitrate transporter 1.14
**Ammonium transporter**							
HORVU.MOREX.r3.2HG0180820	-1.2421	1.1695					Ammonium transporter 1 member 1
HORVU.MOREX.r3.1HG0072140				1.8400			Ammonium transporter 2 member 1
HORVU.MOREX.r3.5HG0530810		5.4330		6.4117		-4.6582	Ammonium transporter 3 member 2
**Nitrate reductase**							
HORVU.MOREX.r3.6HG0541410		-3.6456			-4.3314		Nitrate reductase
**Glutamate synthetase**							
HORVU.MOREX.r3.3HG0280530	1.4136						Glutamate synthase 1
**Glutamine synthetase**							
HORVU.MOREX.r3.1HG0039730	1.1376	-1.0493					Glutamine synthetase
HORVU.MOREX.r3.4HG0395540	1.2124				-1.5744		Glutamine synthetase cytosolic isozyme 1-2
HORVU.MOREX.r3.2HG0202350	-1.8058	-2.3863			-1.0412		Glutamine synthetase leaf isozyme
HORVU.MOREX.r3.6HG0549370					-1.7241		Glutamine synthetase, catalytic domain
HORVU.MOREX.r3.4HG0337240					2.1526		Glutamine synthetase cytosolic isozyme 1-3
HORVU.MOREX.r3.6HG0613270	1.4800	1.7654			1.2366	0.8531	Glutamine synthetase
**Glutamate dehydrogenas**							
HORVU.MOREX.r3.2HG0185380	1.2328						Glutamate dehydrogenase 2
**Asparagine synthetase**							
HORVU.MOREX.r3.1HG0092430		1.7461		2.0034	1.8264		Asparagine synthetase 2
HORVU.MOREX.r3.5HG0441010				1.5588			Asparagine synthetase domain-containing protein 1

HORVU.MOREX.r3.1HG0085120	1.0731	Asparagine synthetase 2
HORVU.MOREX.r3.5HG0465890	-2.3917	Asparagine synthetase

Table S4. Candidate genes related to different N treatment in barley nitrogen metabolism in roots.

Gene ID	W26 Log_2FC			W26 Log_2FC			Annotation
	3d-LN/CK	18d-LN/CK	21d-LN/RN	3d-LN/CK	18d-LN/CK	22d-LN/RN	
**Nitrite transporter**							
HORVU.MOREX.r3.5HG0432940					7.5019		Nitrate transporter 1.2
HORVU.MOREX.r3.2HG0101350					-2.8937		Nitrate transporter 1.10
HORVU.MOREX.r3.2HG0102130					-2.7618		Nitrate transporter 1.2
HORVU.MOREX.r3.6HG0543590		-2.4719					High-affinity nitrate transporter 2.2
HORVU.MOREX.r3.6HG0543570	-2.1595	-3.0985	2.8172		-2.3043		High-affinity nitrate transporter 2.2
HORVU.MOREX.r3.4HG0415780			-1.5407				Nitrate transporter 1.1
HORVU.MOREX.r3.6HG0543410		-7.1164	6.5276	4.6526	2.4549		High-affinity nitrate transporter 2.2
HORVU.MOREX.r3.6HG0543390		-8.9577	3.7417		-3.9596		High-affinity nitrate transporter 2.2
HORVU.MOREX.r3.6HG0543400		-10.9351	6.1277	7.0981			High-affinity nitrate transporter 2.2
HORVU.MOREX.r3.1HG0056390		-4.7883			-4.1137		Nitrate transporter 1.1
HORVU.MOREX.r3.6HG0543380		-5.0743	3.9847				High-affinity nitrate transporter 2.1
HORVU.MOREX.r3.5HG0528300			3.5417	4.8000			High-affinity nitrate transporter-activating protein 2.1
HORVU.MOREX.r3.1HG0056410			1.5363		-1.5680		Nitrate transporter 1.1
HORVU.MOREX.r3.6HG0591170			2.0560				Probable high-affinity nitrate transporter-activating protein 2.2
HORVU.MOREX.r3.2HG0177940					-1.9249		Nitrate transporter 1.2
HORVU.MOREX.r3.6HG0591180			2.7416	2.7956			Probable high-affinity nitrate transporter-activating protein 2.2
HORVU.MOREX.r3.5HG0420230					4.0499		Nitrate transporter 1.10
HORVU.MOREX.r3.2HG0177990					-1.5854		Nitrate transporter 1.2
HORVU.MOREX.r3.3HG0283350			5.3051		3.0526	4.2643	High-affinity nitrate transporter 2.3
HORVU.MOREX.r3.2HG0177980					-3.3222		Nitrate transporter 1.2
HORVU.MOREX.r3.5HG0513990					1.5721		Nitrate transporter 1.3
HORVU.MOREX.r3.6HG0609530			1.7880		-2.6445		Nitrate transporter 1.5

Table S5. The primers used for qRT-PCR.

Gene	Forward	Reverse
HORVU.MOREX.r3.6HG0541410	5' AAGAAGGAGCTCTCCCACGA 3'	5' GCGCAAACGAAGATGTGCTT 3'
HORVU.MOREX.r3.4HG0337240	5' GACACCGAGAAGGAAGGCAG 3'	5' ACTTGCCATTGGAGAGACCG 3'
HORVU.MOREX.r3.6HG0613270	5' GGTGGGTTCAAGGTCATCGT 3'	5' AAGTAGCCCTTGCCGTTCTG 3'
HORVU.MOREX.r3.4HG0386090	5' CTGACGCCTTTACCAGAGCA 3'	5' GACAGGGCAGCTTCGATCTT 3'
HORVU.MOREX.r3.7HG0641120	5' GACGACACGCTACTGAAAGC 3'	5' CCAAAGAACGGCTTCCCTCC 3'
HORVU.MOREX.r3.6HG0543390	5' GGCATCTGTGTCTGGGTCC 3'	5' GACACAAAGGTGGCAAGGG 3'
HORVU.MOREX.r3.1HG0008440	5' CTTCCGTGCTTCAATCCGAC 3'	5' GCGGAAGAAAGAGCTGGGAT 3'
HORVU.MOREX.r3.1HG0051860	5' GACGAGACGATGAGCCGAA 3'	5' GAGTTCGCTCTTGTTGGCGA 3'
HORVU.MOREX.r3.2HG0202910	5' GCTCATCGTGCAGTACCTCG 3'	5' CCTTGATCCACGAGGCAAAGA 3'
HvACTIN	5' GCCGTGCTTTCCCTCTATG 3'	5' GCTTCTCCTTGATGTCCCTTA 3'

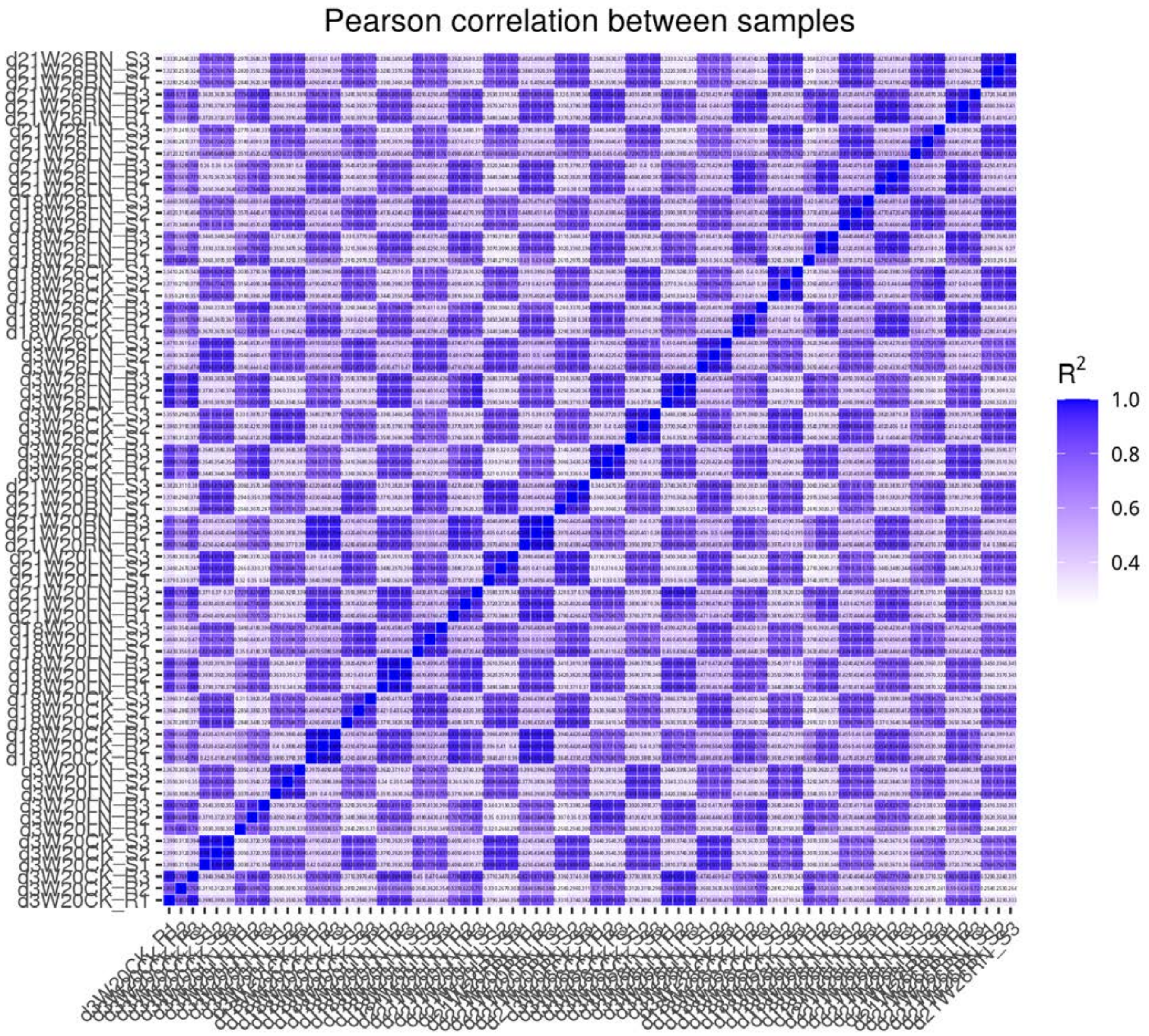

Figure S1. Pearson correlation between samples for RNA-Seq."d3W26_S" represents the leaves sampled by W26 on the 3rd day. "d18W26_S" represents the leaves sampled by W26 on the 18th day. "d21W26_S" represents the leaves sampled by W26 on the 21st day. "d3W26_R" represents the roots sampled by W26 on the 3rd day. "d18W26_S" represents the roots sampled by W26 on the 18th day. "d21W26_S" represents the roots sampled by W26 on the 21st day.

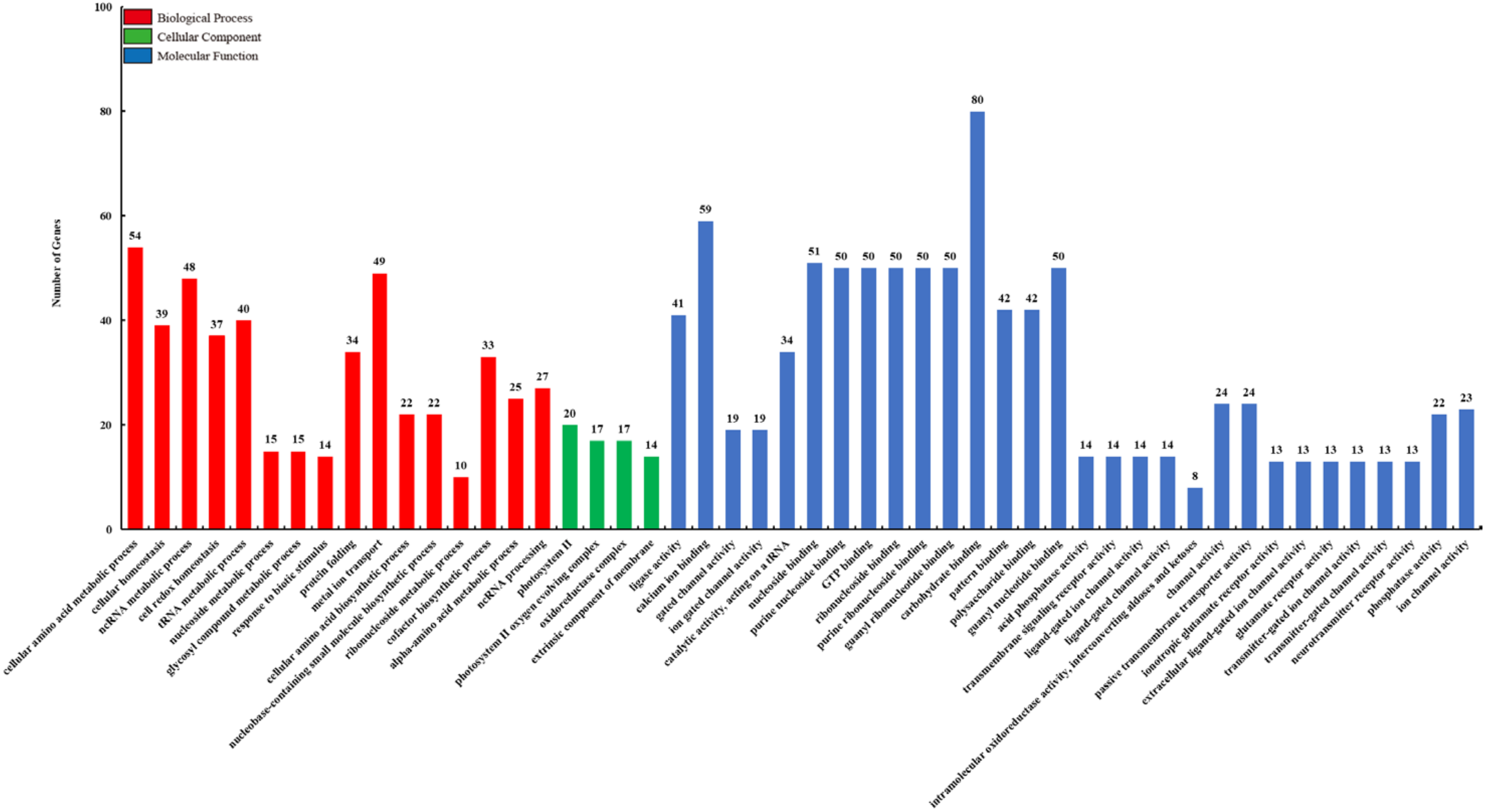

Figure S2. GO annotation of DEGs in leaves for W26.

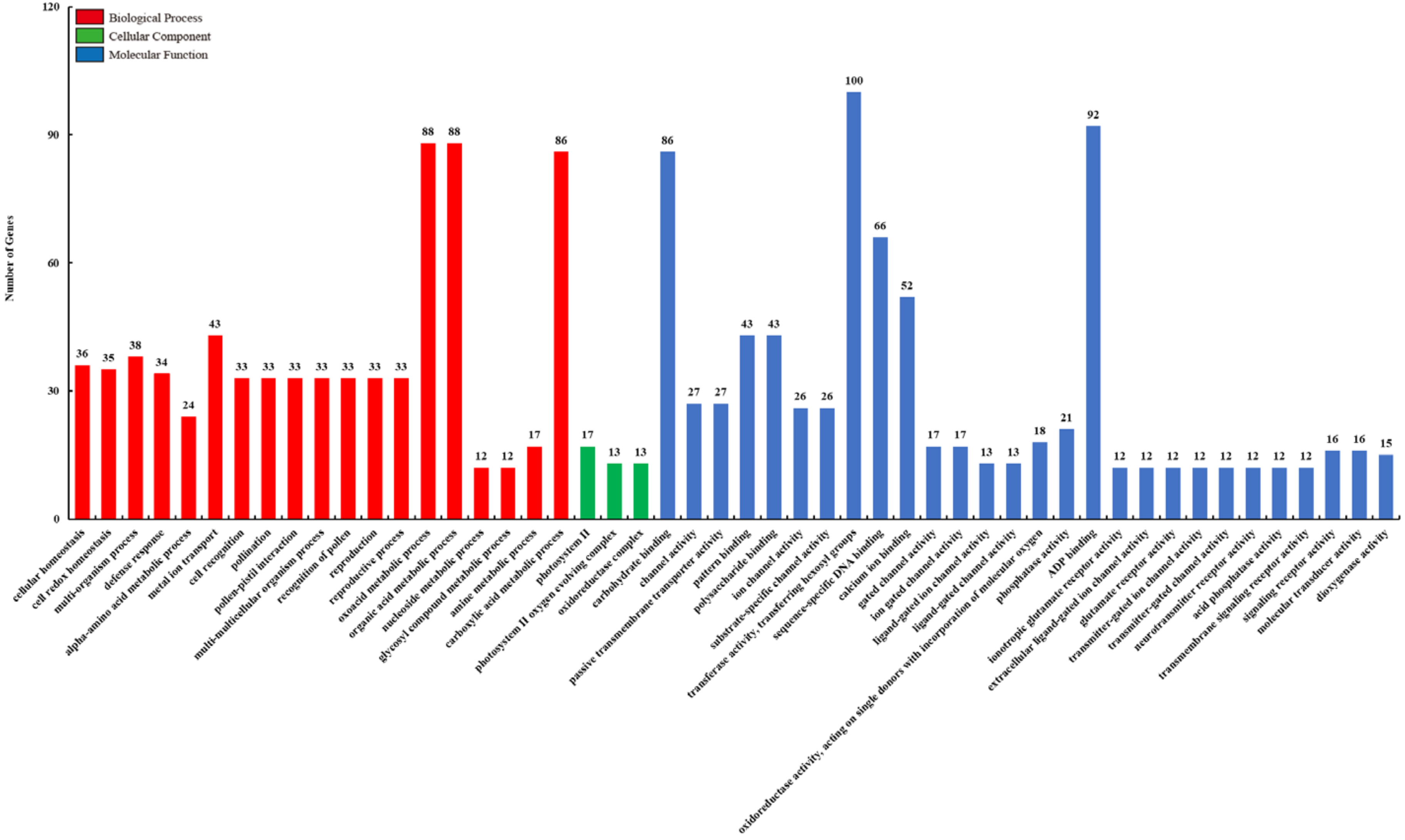

Figure S3. GO annotation of DEGs in leaves for W20.

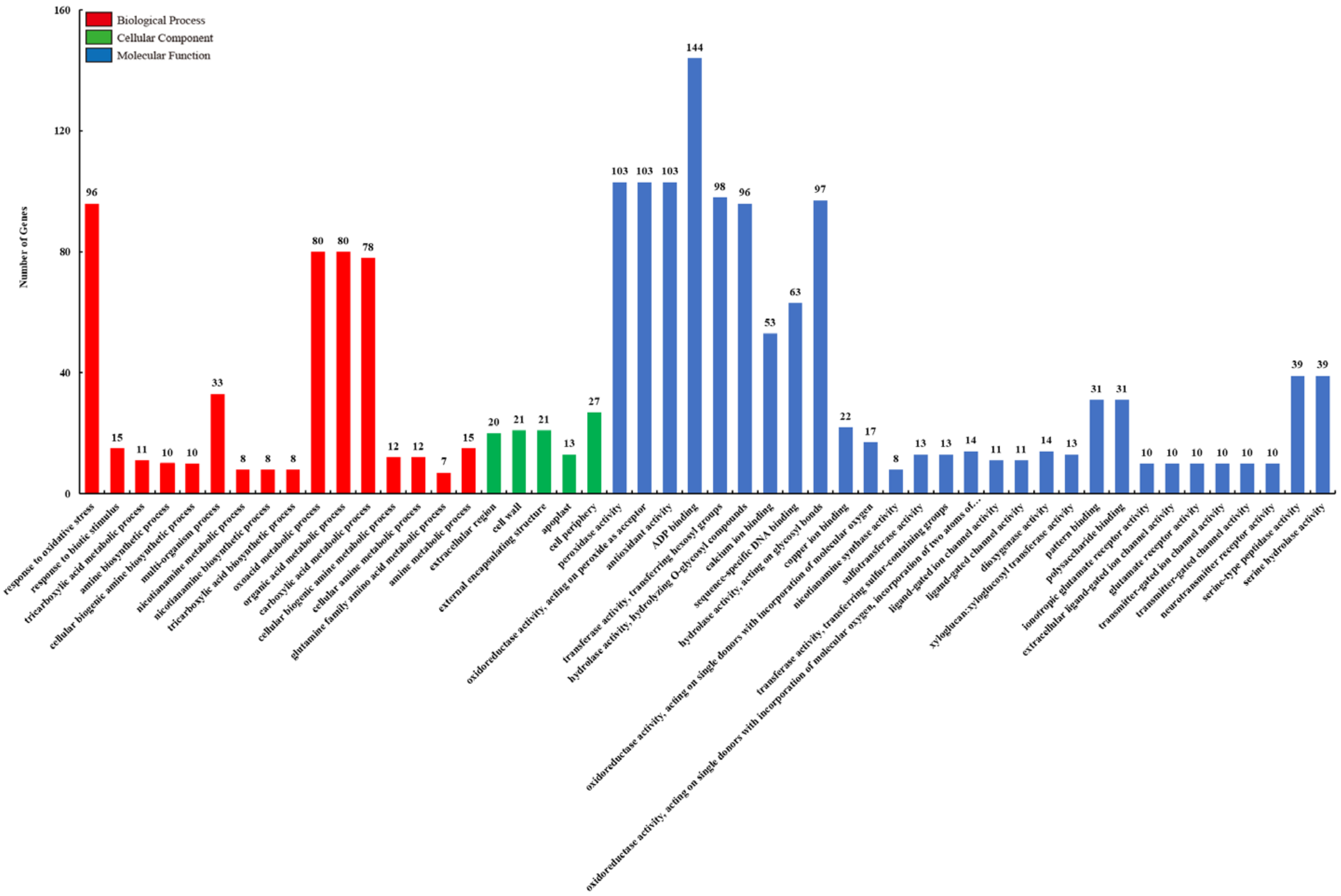

Figure S4. GO annotation of DEGs in roots for W26.

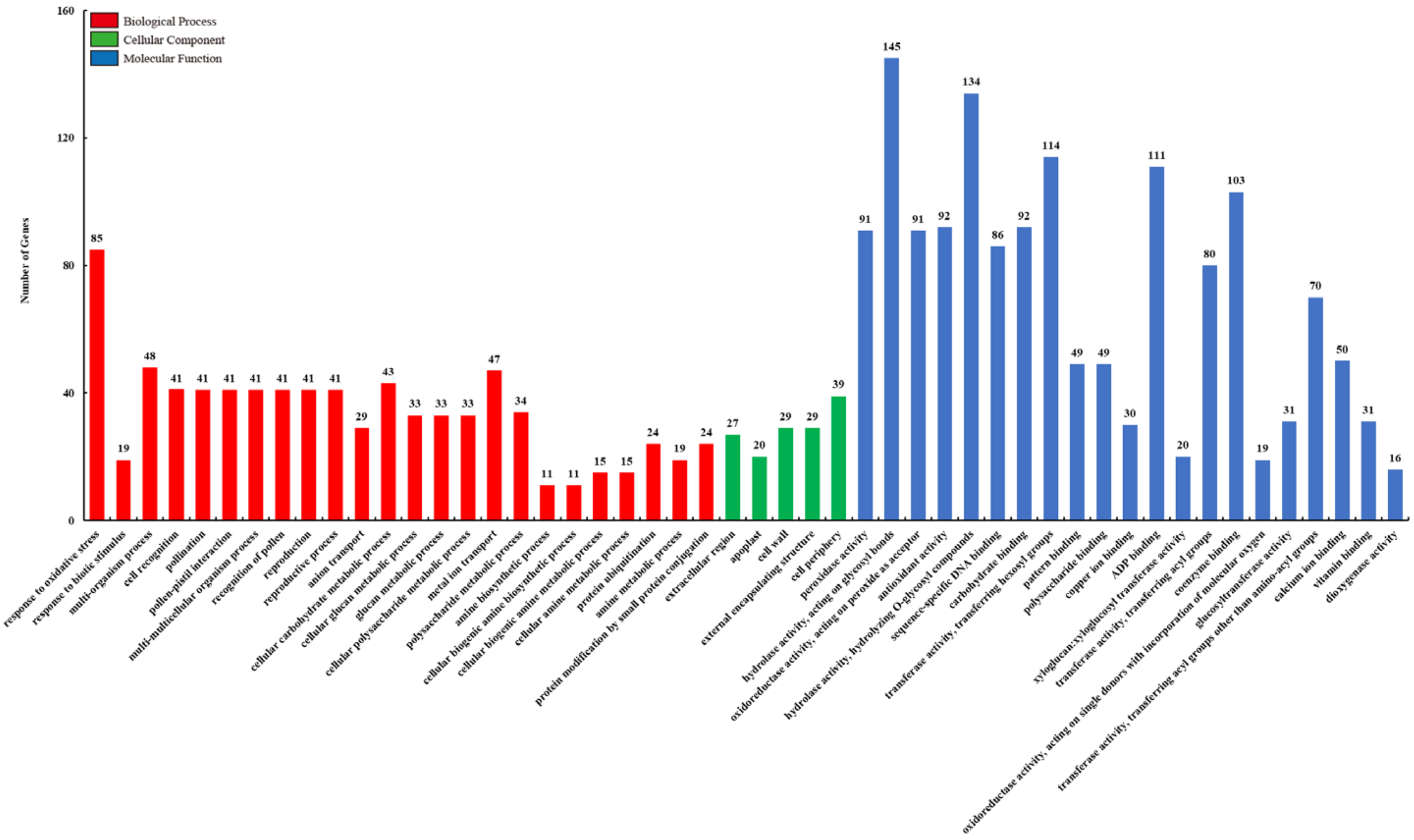

Figure S5. GO annotation of DEGs in roots for W20.

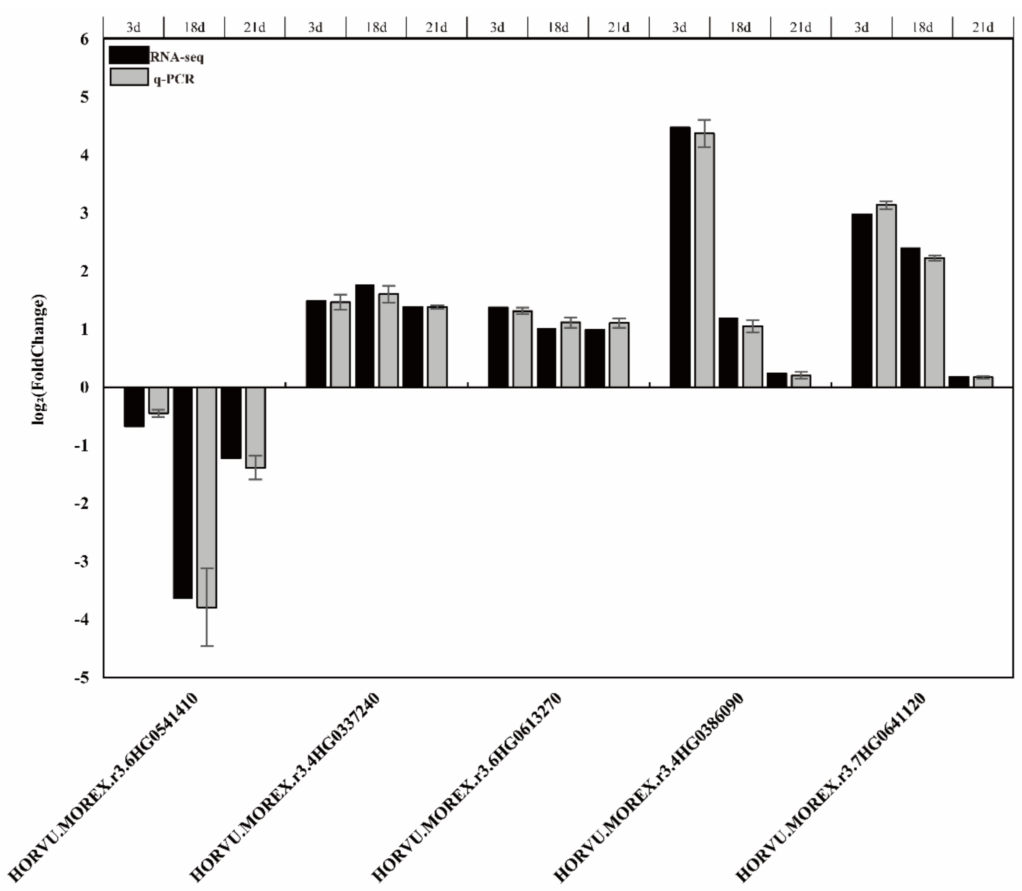

Figure S6. qRT-PCR validation of the barley in leaves for W26 differentially expressed genes in different nitrogen treatments.

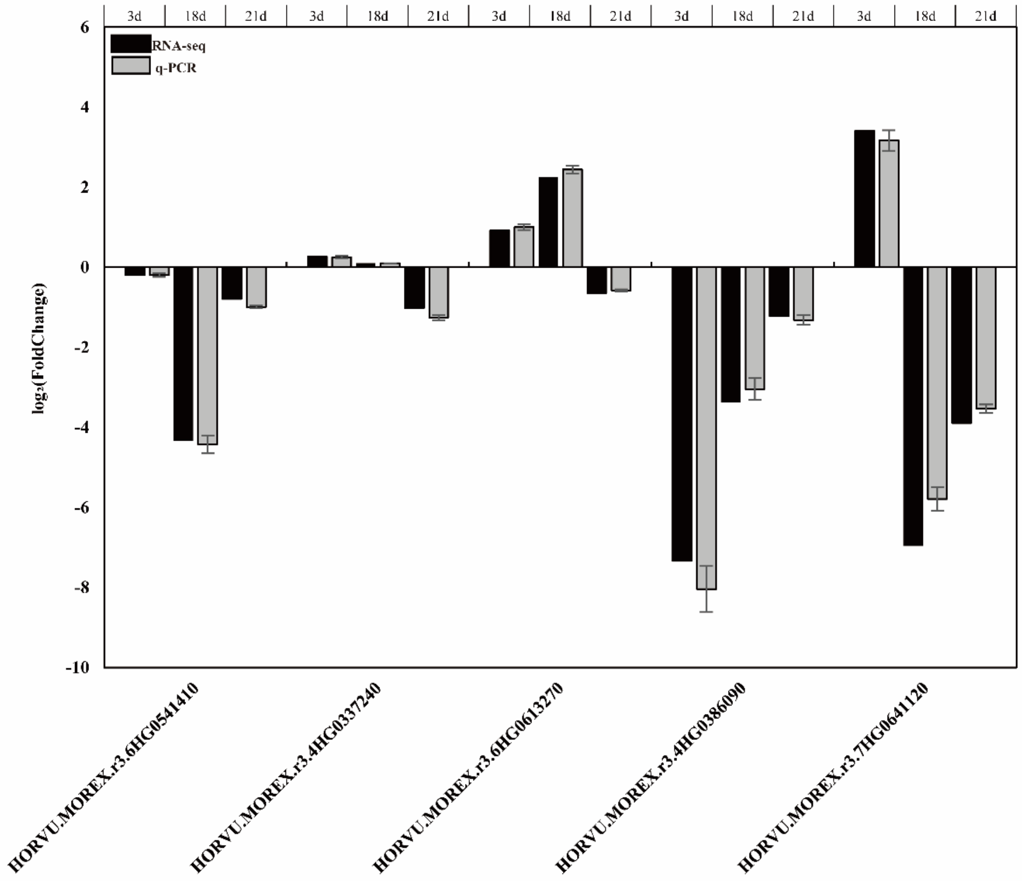

Figure S7. qRT-PCR validation of the barley in leaves for W20 differentially expressed genes in different nitrogen treatments.

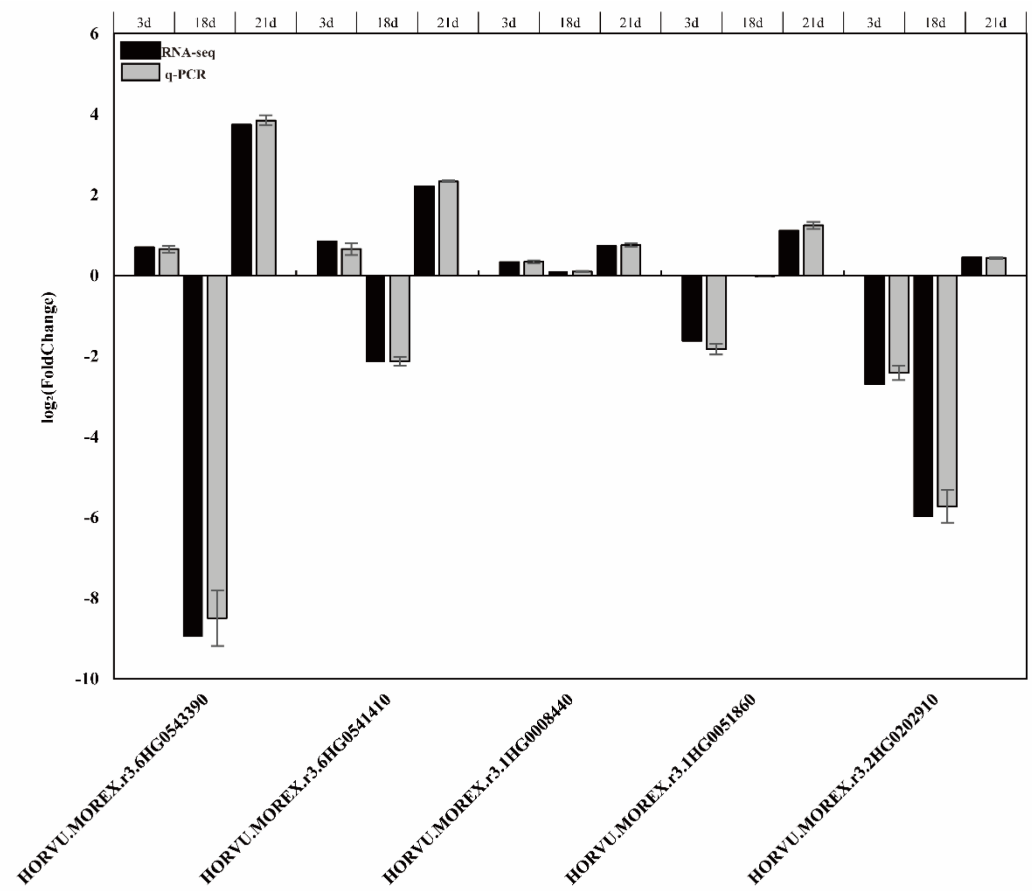

Figure S8. qRT-PCR validation of the barley in roots for W26 differentially expressed genes in different nitrogen treatments.

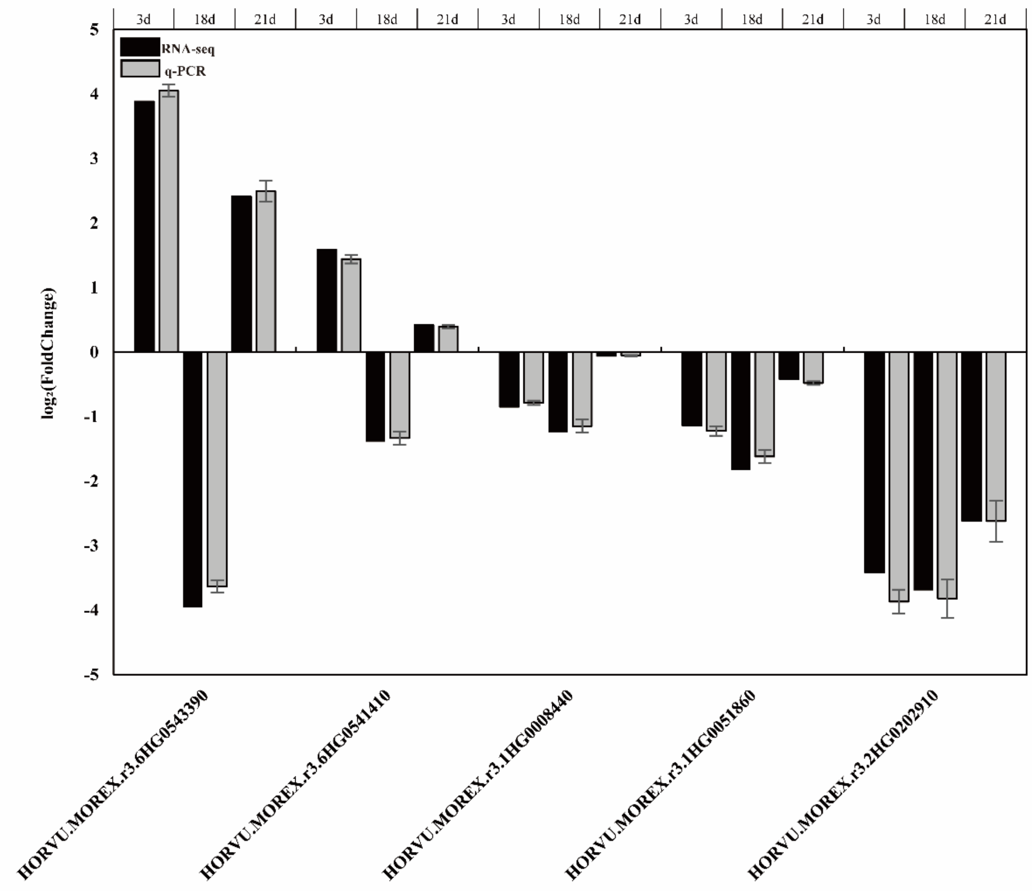

Figure S9. qRT-PCR validation of the barley in roots for W20 differentially expressed genes in different nitrogen treatments.
